# Immune dysregulation and gut microbiota: Connection to health and disease development

**DOI:** 10.4103/NRR.NRR-D-25-00244

**Published:** 2025-09-29

**Authors:** Ana Paula de Araújo Boleti, Pedro Henrique de Oliveira Cardoso, Breno Emanuel Farias Frihling, Luiz Filipe Ramalho Nunes de Moraes, Ellynes Amancio Correia Nunes, Lincoln Takashi Hota Mukoyama, Maria Eduarda Freitas Biembengute, Vívia Cleisla Bezerra de Melo, Marcos Fernandes Morales, Alinne Pereira de Castro, Ludovico Migliolo

**Affiliations:** 1 S-Inova Biotech, Programa de Pós-Graduação em Biotecnologia, Universidade Católica Dom Bosco, Campo Grande, MS, Brazil; 2 Programa de Pós-graduação em Bioquímica, Universidade Federal do Rio Grande do Norte, Natal, Brazil; 3 Programa de Pós-graduação em Biologia Celular e Molecular, Universidade Federal da Paraíba, João Pessoa, Brazil

**Keywords:** dysbiosis, gastrointestinal disorders, immune response, inflammation, microbiota, nerve regeneration, neurological diseases

## Abstract

Neuroinflammation is a key pathophysiological mechanism in various neurological diseases, such as Alzheimer’s disease and neuropsychiatric disorders. This review article analyzes the role of inflammatory mediators and their signaling pathways in the pathogenesis of neurodegenerative diseases, focusing on the molecular mechanisms that promote neurodegeneration and hinder neural regeneration. Recent evidence suggests that alterations in inflammatory processes in the brain are associated with cognitive impairment, progressive neurodegeneration, and impaired synaptic plasticity. Activation of Toll-like receptors triggers an inflammatory cascade involving pro-inflammatory cytokines such as interleukins and tumor necrosis factor alpha, which significantly contributes to neuronal dysfunction and cell death. Furthermore, we are investigating how these neuroinflammatory processes interfere with neural regeneration mechanisms and the maintenance of central nervous system homeostasis. Neural biomarkers associated with inflammation are emerging as potential diagnostic tools and therapeutic targets for neurological diseases, particularly anxiety disorders and Alzheimer’s disease. Both experimental and clinical studies suggest that interventions aimed at modulating neuroinflammation could be a promising therapeutic approach to promoting neuroprotection and stimulating neural regeneration. This review summarizes the latest research on the molecular mechanisms of neuroinflammation and its effect on brain function. It also highlights potential neuroprotective and regenerative strategies for treating neurological diseases.

## Introduction

The gut microbiota (GM) is a complex community of microorganisms living in the digestive tract of humans and animals, including insects. It plays a crucial role in modulating physiological processes such as gastrointestinal motility, secretion, maintenance of epithelial barrier integrity, and communication between the gut and the central nervous system (Bhattarai et al., 2017; Saffouri et al., 2019). GM includes diverse microbial species, including bacteria, archaea, fungi, and viruses (Laterza et al., 2016; Passos and Moraes-Filho, 2017; Rinninella et al., 2019).

**Figure 1:**
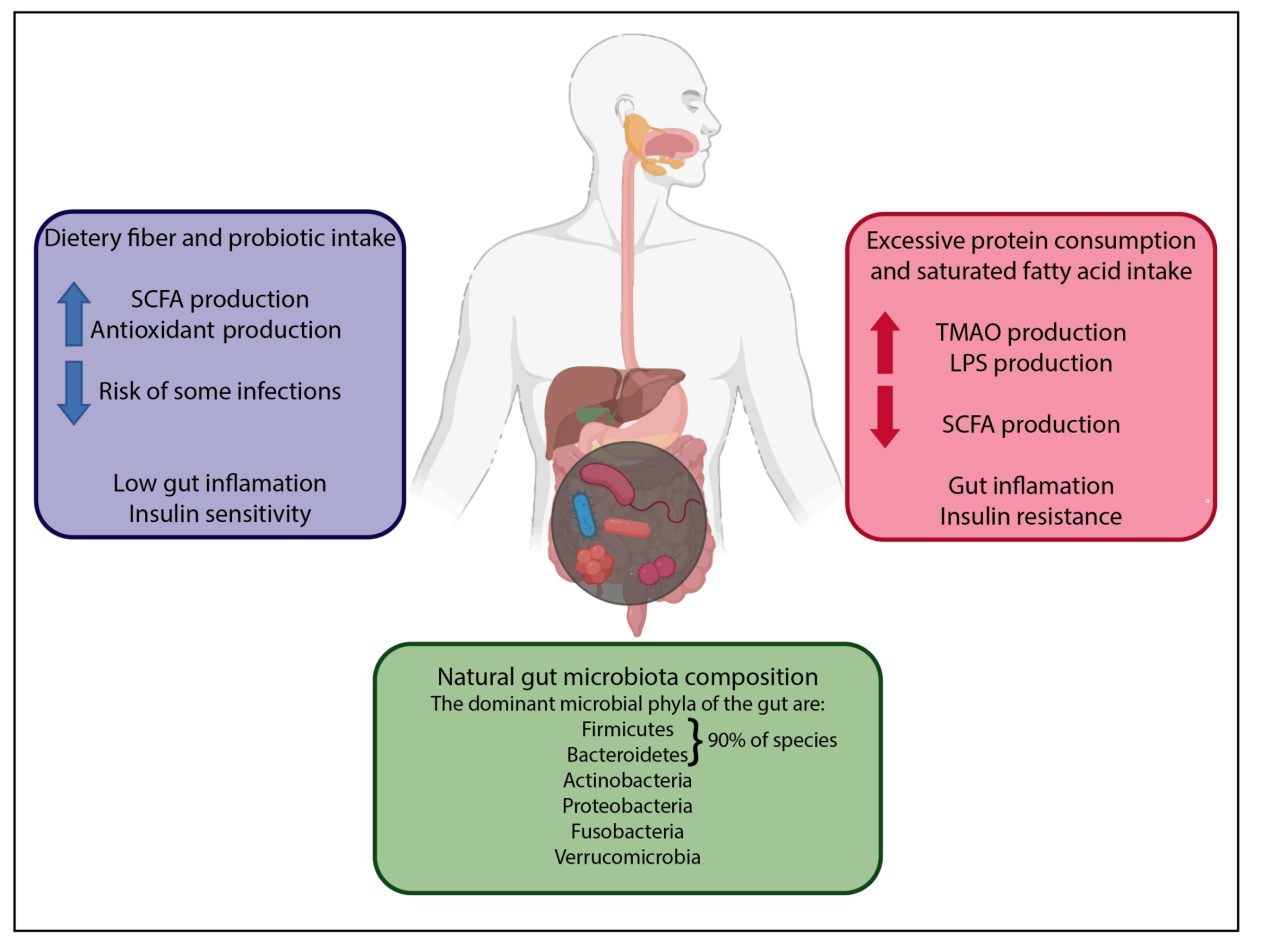
Overview of the impact of diet on gut microbiota and metabolism. Created with BioRender.com. LPS: Lipopolysaccharide; SCFA: short-chain fatty acids; TMAO: trimethylamine N-oxide.

The dominant microbial phyla in the gut include Firmicutes, Bacteroidetes, Actinobacteria, Proteobacteria, Fusobacteria, and Verrucomicrobia, of which Firmicutes and Bacteroidetes together account for 90% of the total microbiota (Arumugam et al., 2011; Gomaa, 2020). The Firmicutes phylum comprises over 200 genera, including *Lactobacillus*, *Bacillus*, *Clostridium*, *Enterococcus*, and *Ruminococcus*, with *Clostridium* species representing 95% of the phylum. Bacteroidetes is predominantly composed of the genera *Bacteroides* and *Prevotella*. The Actinobacteria phylum is less abundant and is mainly represented by the genus Bifidobacterium (Arumugam et al., 2011). The Firmicutes/Bacteroidetes ratio is a key indicator for finding gut microbiota imbalances (Chen et al., 2021). Additionally, microbial abundance, diversity, and uniformity are crucial parameters in assessing a healthy gut microbiota composition (**Figure 1**).

Several factors can influence the composition and function of the intestinal microbiota, including host genetics, diet, age, birth mode, and antibiotic use (Mesa et al., 2020; Ramirez et al., 2020; Singh et al., 2021). When a GM imbalance occurs, it alters both its structure and function, contributing to the onset and progression of certain diseases (**Figure 1**). In recent years, advances in molecular biology, genomics, bioinformatics, and high-throughput sequencing technologies have significantly enhanced GM research.

The GM is separated from the host’s internal environment by a single layer of epithelial cells, presenting a unique challenge to the immune system. The immune system has evolved to recognize microbial agents as potential pathogens, making continuous interactions with the microbiota essential for immune regulation. Conversely, immune responses to microbiota also influence its ecology, shaping both its composition and function (Al Bander et al., 2020).

Extensive research has demonstrated that GM imbalances and their metabolites play a crucial role in maintaining intestinal homeostasis and influencing the development of various diseases, including neurodegenerative, cardiovascular, metabolic, and gastrointestinal disorders. This review provides a comprehensive discussion on gut dysbiosis and its impact on the human immune system. Additionally, we explore the relationship between intestinal dysbiosis and gastrointestinal and neurological pathologies, as well as its role in skin diseases.

## Search Strategy

The search strategy for this narrative review comprehends using the PubMed database to find article published after 2000 using the keywords: dysbiosis; gastrointestinal disorders; immune response; inflammation; microbiota; nerve regeneration; neurological diseases. All articles find in the search were only included if it fits in the scope of the review.

## General Gut Dysbiosis

Intestinal dysbiosis is a clinical condition defined by an imbalance in the gut microbiota (Weiss and Hennet, 2017; Al Bander et al., 2020; Martinez et al., 2021). This condition is believed to be associated with the development of several immune-mediated disorders, including inflammatory bowel disease (IBD), rheumatoid arthritis, diabetes mellitus, multiple sclerosis, and systemic lupus erythematosus, among others (Horta-Baas et al., 2017; Principi et al., 2018; Martinez et al., 2021; Bielka et al., 2022; Cantoni et al., 2022). IBD, which includes Crohn’s disease (CD) and ulcerative colitis (UC), is a chronic inflammatory condition that is becoming increasingly prevalent worldwide. It is thought to result from an inflammatory response of the intestinal mucosa. Chronic inflammation of the digestive tract is often observed in patients with IBD, alongside reduced microbial diversity (Principi et al., 2018).

The gut hosts a unique and dynamic microbiome, constantly exposed to external stimuli such as diet, antibiotics, xenobiotics, and pathogens (Francino, 2015). Digestible and non-digestible carbohydrates, proteins, fats, polyphenols, prebiotics, and probiotics can induce changes in the microbiota, influencing the host’s immune and metabolic markers (Singh et al., 2017). This suggests a close relationship between the gut microbiome, health, and diet, where improvements in health can be modulated through dietary changes affecting the microbiota.

The gut microbiota plays a vital role in the absorption, storage, and expenditure of energy from the diet. Studies have shown that the absorption of fats, simple carbohydrates, and proteins begins in the duodenum and continues through the jejunum. The distal part of the small intestine is primarily responsible for absorbing bile acids and vitamin B12, while the colon absorbs water, electrolytes, and short-chain fatty acids (SCFAs) produced by bacterial fermentation (Kiela and Ghishan, 2016; Koh et al., 2016).

Changes in gut microbiota can lead to symptoms of functional gastrointestinal disorders through fermentation of ingested food, modulating bile acid metabolism, and altering intestinal immune function, visceral motility, and sensation (Wei et al., 2021). Alterations in the microbiota in gastric fluid have been reported in patients with functional dyspepsia, and fecal dysbiosis has been observed in individuals with irritable bowel syndrome and functional constipation (Ford et al., 2020).

The gut microbiota works in conjunction with host defenses and the immune system to protect against pathogen colonization and invasion (Carding et al., 2015). It also performs essential metabolic functions, such as acting as a source of vitamins and nutrients, aiding in energy extraction, and producing SCFAs and amino acids from food. Additionally, the host depends on its gut microbiota for several vital functions, including digestion, nutrient synthesis, immune system development, and neurotransmitter production, thereby contributing to overall health (Carding et al., 2015; Yoo et al., 2020). Although it is challenging to fully delineate the precise impact of gut microbiota on human health and disease, it is well established that microbiota play a key role in nutrient and drug absorption, vitamin synthesis (e.g., B12 and K), and endocrine regulation, which ultimately affects metabolism (Martinez et al., 2021).

SCFAs (acetate, propionate, and butyrate) are produced by bacterial fermentation of dietary fibers and exert neuroprotective effects, preserving the integrity of the blood–brain barrier (Zhaoying Li et al., 2023; Thapa et al., 2024). Butyrate, for instance, functions as a histone deacetylase inhibitor, thereby promoting histone acetylation and the expression of pro-regenerative genes, including brain-derived neurotrophic factor (BDNF), in neurons and glial cells (Chriett et al., 2019; Maejima et al., 2020). BDNF has been shown to regulate synaptic plasticity and axonal regeneration, thereby promoting neuronal survival and axon growth following injury (Liu et al., 2017).

Tryptophan metabolites, such as indole-3-propionic acid, have been shown to modulate the aryl hydrocarbon receptor pathway, which is involved in reducing neuroinflammation and promoting neural repair (Li et al., 2025). Intestinal dysbiosis has been demonstrated to alter DNA methylation patterns and histone modification in neurons, thereby impacting the expression of genes associated with neuroprotection (D’aquila et al., 2020). Butyrate, by inhibiting histone deacetylases, has been shown to increase the availability of acetyl-CoA, thus favoring the transcription of anti-apoptotic genes (e.g., Bcl-2) and neural growth factors (Shukla and Tekwani, 2020). Furthermore, microbiota has been demonstrated to regulate the production of microRNAs (e.g., miR-132) that modulate the expression of proteins involved in axonal integrity, such as GAP-43 (Li et al., 2024). Chronic stress induces persistent epigenetic modifications, including hypermethylation of the BDNF promoter and changes in the expression of microRNAs that regulate neuronal survival, such as miR-132 (Won and Kim, 2020).

Studies on animal models have demonstrated that regular aerobic exercise (30 minutes a day for 6 weeks) can significantly boost the expression of BDNF in the hippocampus. This occurs through the demethylation of its promoter and the acetylation of histones H3K9/K14. The outcome is an increase in synaptic density and enhanced protection against neuronal apoptosis caused by oxidative stress (Gomez-Pinilla et al., 2011).

## Gut Dysbiosis Impact on the Immune System

The immune system is directly involved in modulating the gut microbiota based on the type of food consumed, contributing to tissue repair and defending against pathogens, thereby ensuring proper organ function (Shi et al., 2017). The gastrointestinal tract (GIT) harbors a complex and dynamic matrix of antigens derived from food, commensals, and pathogens, presenting significant challenges for the intestinal immune system (Guo et al., 2021). To address these challenges, a specialized set of immune responses has evolved to protect the gut. The lamina propria of the intestine, particularly in the small intestine, contains the highest concentration of antibody-secreting cells in the body, accounting for more than 80%. These antibody-secreting cells secrete antibodies into the luminal gut, where they bind to opportunistic pathogens, limiting their invasion (Peterson and Artis, 2014; Honda and Littman, 2016; Lueschow and McElroy, 2020).

Antibodies also bind to commensals, regulating their composition, distribution, and pro-inflammatory activity. Generally, immunoglobulin A (IgA) shows a hyporesponsive reaction to commensals but actively responds to pathogens (Stolfi et al., 2022). Of the five known mammalian antibody isotypes, IgA, IgM, and IgG can be secreted into the luminal gut, with IgA making up the largest proportion (Guo et al., 2021). IgA serves as the first line of defense, protecting intestinal epithelial cells from toxins, pathogens, and commensals. In addition to the intestine, IgA-secreting antibody-producing cells can also be detected in the salivary glands, lungs, mammary glands, liver, and bone marrow (Forbes et al., 2008). In healthy gut microbiota, IgA plays a crucial role in controlling microbial composition and promoting host health by protecting against opportunistic pathogens, modulating the immune system, and aiding the fermentation of indigestible polysaccharides to produce SCFAs (Peterson and Artis, 2014; Wells et al., 2017).

The GIT also contains a variety of cells that perform specialized functions, including enterocytes, which are responsible for nutrient absorption; goblet cells that regulate mucus secretion; enteroendocrine cells that contribute to hormonal regulation for gastrointestinal homeostasis; and Paneth cells that secrete antimicrobial granules and peptides such as defensins, lysozymes, and phospholipases A2 (Peterson and Artis, 2014; Takiishi et al., 2017; Lueschow and McElroy, 2020; Stolfi et al., 2022).

GIT is an organ characterized by extensive microbial diversity and an abundance of B and T cells, which are essential for maintaining homeostasis with the microbiota. Th17 cells, for instance, play a central role in preserving the intestinal barrier by producing cytokines such as IL-17A, IL-17F, and IL-22, which enhance epithelial integrity and stimulate antimicrobial responses (Martinez-Guryn et al., 2019; Shim et al., 2023).

In addition to Th17 cells, other cell types such as innate lymphoid cells, natural killer cells, and γδ T cells also produce IL-17 and IL-22, contributing to the early defense against infections, such as those caused by *Citrobacter rodentium* (Hall et al., 2013). Th17 cells maintain a feedback loop with the microbiota, regulating adaptive immune responses (Heidari et al., 2024). RORγt deficiency, an essential transcription factor for these cells, disrupts intestinal homeostasis and increases susceptibility to injury and inflammation (López-Fandiño et al., 2023).

The microbiota, including segmented filamentous bacteria, is vital for the generation of Th17 cells. While segmented filamentous bacteria provides protection against infections, it can also induce susceptibility to autoimmune diseases (Flannigan and Denning, 2018). The balance between protection and autoimmune risk mediated by Th17 cells and the microbiota still requires further investigation.

IL-17 and IL-22 signaling protects against dextran sulfate sodium-induced colonic epithelial damage by promoting tissue repair and maintaining homeostasis. Mice deficient in these pathways exhibit increased mucosal damage and delayed recovery, whereas exogenous administration of IL-22-Fc restores epithelial function (Weaver et al., 2013; Liu et al., 2021c).

Innate lymphoid cells are primary sources of IL-22 during acute injury, while the absence of IL-23 increases regulatory T-cells and reduces inflammation (Ignacio et al., 2017). Despite their protective role, Th17 cells can mediate colitis, highlighting a functional duality between protection and pathogenicity that remains to be elucidated (Wen et al., 2024).

Microbiota exists throughout an individual’s life, influenced by factors such as birth method, maternal diet, environmental exposures, and the host’s genetics (Takiishi et al., 2017; Tanaka and Nakayama, 2017; Lueschow and McElroy, 2020). Consequently, microbiota is essential not only for protecting the GIT but also for promoting homeostasis, energy expenditure, and the establishment of both innate and adaptive immunity. This is made possible by the harmonious relationship between adjacent organs and the microbiota, each of which has distinct compositions that contribute to eubiosis (Brown et al., 2012; Álvarez et al., 2021).

Gut dysbiosis, a condition that can gradually worsen without clear symptoms, can be observed in cases such as irritable bowel syndrome, where dysfunction is triggered by food dysregulation, psychological factors, hypersensitivity of nerve receptors in the intestinal wall, elevated levels of neurotransmitter production, and disruptions in the development of lymphoid tissues (Lobo and Miranda, 2010; Canakis et al., 2020; Suárez et al., 2021).

Gut dysbiosis has been demonstrated to disrupt immune-microglia crosstalk, resulting in aberrant glial activation and impaired myelin repair. For instance, bacterial lipopolysaccharide (LPS) from Gram-negative pathogens has been demonstrated to trigger Toll-like receptor 4 (TLR4) signaling in microglia, resulting in the release of pro-inflammatory cytokines (e.g., interleukin [IL]-17, tumor necrosis factor alpha [TNF-α]) that, in turn, inhibit oligodendrocyte progenitor cell differentiation (Rothhammer et al., 2018; Sanmarco et al., 2021). Conversely, SCFAs derived from fiber-fermenting bacteria, such as Clostridia, have been observed to promote the expansion of regulatory T-cells, which in turn suppress neuroinflammation and facilitate remyelination in experimental autoimmune encephalomyelitis models (Berer et al., 2017; Duscha et al., 2020). These findings position the gut microbiota as a key regulator of glial-immune interactions in demyelinating diseases.

Recent research identifies *Bacteroides fragilis* as a critical modulator of microglial homeostasis; its polysaccharide A metabolite induces anti-inflammatory microglial phenotypes and enhances oligodendrocyte progenitor cell maturation in murine models of multiple sclerosis (Erturk-Hasdemir et al., 2021). Dietary interventions (e.g., high-fiber diets) that elevate butyrate levels similarly reduce microglial activation and improve myelin repair in experimental autoimmune encephalomyelitis (Erturk-Hasdemir et al., 2021). It is therefore hypothesized that targeting gut-immune pathways may offer a novel strategy for the mitigation of glial dysfunction in neuroinflammatory disorders.

### Gut dysbiosis and inflammatory process

Both genetic and specific environmental factors are known to contribute to the disruption of the intestinal barrier and promote intestinal dysbiosis (Nibali et al., 2014). Specifically, impaired expression of genes related to cellular integrity, junctional complexes, mucus production and secretion, Paneth cell function, pathogen detection, production of ROS, response to xenobiotics, and IgA secretion significantly compromises the integrity of the intestinal epithelial barrier and its protective function (Stolfi et al., 2022). Environmental factors, such as bacterial infections, exposure to drugs (e.g., antibiotics) following pathogen infections or other illnesses, and an increased intake of fat, sugars, and ethanol at the expense of fruits and vegetables, have been shown to affect host microbiota composition and metabolic activities. These changes lead to a loss of commensals and overgrowth of pathogens (Chan et al., 2013).

In dysbiosis, a range of pathogenic bacteria can proliferate, establishing colonies and triggering an infectious process (Belizário et al., 2018; Gagliardi et al., 2018). The presence of these pathogens leads to the release of exotoxins, such as LPS, a key component of the cell wall of Gram-negative bacteria (Jacobs et al., 2020). LPS and other pathogen-associated molecular patterns can activate Toll-like receptors (TLRs), present on the membranes of various cells, particularly those involved in immune defense in the GIT (Buchholz and Bauer, 2010; Tang et al., 2021).

TLRs are a family of transmembrane proteins that serve as pattern recognition receptors (Kawai and Akira, 2011). These proteins participate in recognizing pathogen-associated molecular patterns and initiating a cascade of chemical reactions that induce a tailored immune response depending on the type of pathogen (Moriyama and Nishida, 2021). Each pathogen type has specific TLRs that recognize distinct molecular patterns, such as TLR4 for LPS (Tam et al., 2021), TLR13 for ribosomal RNA (Wang et al., 2016), TLR1, 2, and 6 for lipoproteins (Korppi et al., 2018), and TLR5 for flagellins (Vijay-Kumar et al., 2008). TLR9 detects DNA patterns from viruses, bacteria, parasites, and tumors (Yu et al., 2017; Alzahrani, 2020; Naing et al., 2021).

Upon pathogen detection, TLRs activate through interactions with dimers in specific ectodomains on their extracellular region, classified as either homodimers (TLRs 3, 4, 5, 7, 8, and 9) or heterodimers (TLRs 1, 2, and 6) (Vidya et al., 2018). A well-known example is TLR4, which forms a homodimer and interacts with two LPS molecules (Ciesielska et al., 2021).

These receptors are found both intra- and extracellularly, on the cytoplasmic membrane and within endosomes (Akira and Takeda, 2004; Kawai and Akira, 2011; Vidya et al., 2018; Tam et al., 2021). When a pathogen infects the human body, the presence of pathogen-associated molecular patterns and other endogenous ligands is detected through the interaction of the TLR4 receptor with the MD2 protein (Barker and Weiss, 2019). Upon activation, two primary pathways can be triggered: the TRIF-dependent pathway (TIR-domain-containing adapter-inducing interferon-β) and the MYD88-dependent pathway (Myeloid differentiation primary response 88), both of which are proteins on the inner membrane face, exposed to the cytoplasm (Kuzmich et al., 2017; Lv et al., 2020; Kobayashi et al., 2022).

**Figure 2:**
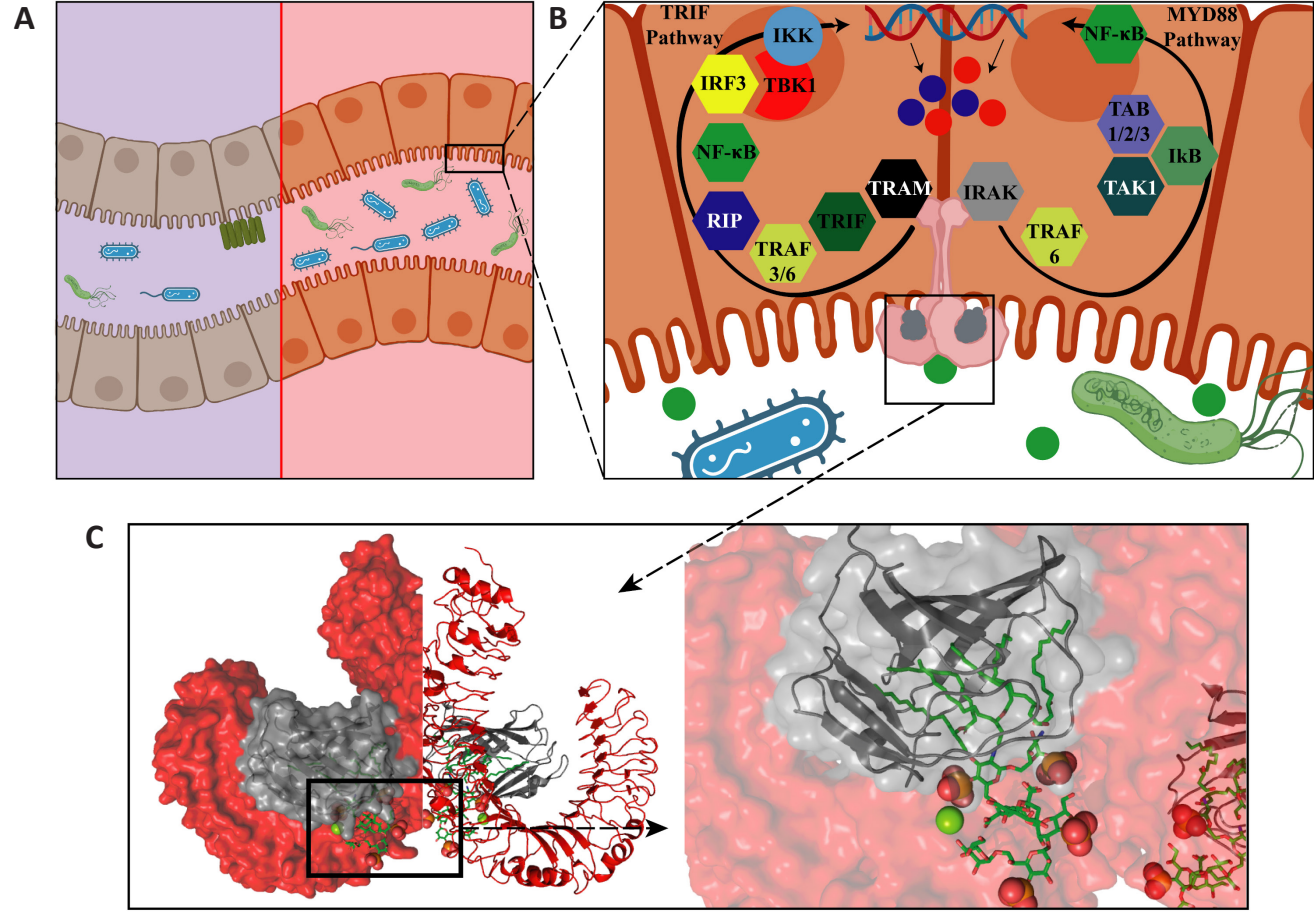
Dysbiosis and inflammatory process. (A) Intestinal microbiota imbalance process in the eubiosis and dysbiosis stages. (B) Recognition of bacterial LPS antigens and activation of pro-inflammatory cytokine and interferon expression pathways in intestinal lumen cells. (C) Molecular interaction complex with LPS antigen (green) mediated by the MD2 coupler (gray) to the TLR4 receptor (red). Created with BioRender.com. IKK: IκB kinase; IRAK: interleukin-1 receptor-associated kinase; IRF3: interferon regulatory factor 3; LPS: lipopolysaccharide; NF-κB: nuclear factor kappa-light-chain-enhancer of activated B cells; RIP: receptor-interacting protein; TAB1/2/3: TAK1-binding protein 1/2/3; TAK1: transforming growth factor-β-activated kinase 1; TBK1: TANK-binding kinase 1; TRAF 3/6: TNF receptor-associated factor 3/6; TRAF6: TNF receptor-associated factor 6; TRAM: TRIF-related adapter molecule; TRIF: TIR-domain-containing adapter-inducing interferon-β.

In the TRIF-dependent pathway, the activation of TLR4 recruits TRIF-related adaptor molecule proteins, which are essential for pathway activation. This leads to conformational changes that enable TRIF to interact with TNF receptor-associated factors (TRAF3 and TRAF6), further interacting with receptor-interacting serine-threonine kinase 1 (Rowe et al., 2006; Lutgens et al., 2010; Liu et al., 2019; Tam et al., 2021). These interactions activate nuclear factor kappa B (Li et al., 2021), while IRF3 is activated through TBK1 and IKK complex stimulation, resulting in the synthesis of type 1 interferons and pro-inflammatory cytokines (Liu et al., 2012; Roy et al., 2014; Ahmad et al., 2016; Tam et al., 2021; **Figure 2**).

The MYD88-dependent pathway, conversely, involves interactions between MYD88 and TLR4, which activate interleukin-1 receptor-associated kinases and TRAF6 complexes (Muroi and Tanamoto, 2012; Mishima et al., 2019). These interactions initiate a phosphorylation cascade, signaling through TAK1, TAB1/2/3, MAP kinases, and IκB complexes (Cronin et al., 2012; Zhang et al., 2017), resulting in nuclear factor kappa B translocation to the nucleus and the activation of pro-inflammatory cytokine and chemokine genes (Lu et al., 2008; Tam et al., 2021; **Figure 2**).

When the immune system is in homeostasis with the body, it can influence the colonization of beneficial bacteria that make up the healthy human microbiota (Álvarez et al., 2021). However, in cases of low immunity or infections from the environment, opportunistic pathogens such as *Escherichia coli*, *Klebsiella pneumoniae*, *Clostridioides difficile*, *Salmonella spp.*, and *Staphylococcus* *aureus* may colonize the GIT (Arboleya et al., 2015; Garfias-López et al., 2018; Rodríguez et al., 2020; Rogers et al., 2021).

Beyond the TLR pathway, the immune system plays a pivotal role in microbiota control via innate immunity, tolerating commensal bacteria that reside in the GIT while combating pathogens (Thaiss et al., 2016). The epithelial barrier itself limits microbial spread, preventing systemic infections, and several cell types interact with the microbiota to maintain homeostasis through both endogenous and exogenous regulation connected to the nervous system (Wells et al., 2017).

These cells include enterocytes (responsible for nutrient absorption and comprising 90%–95% of the intestinal cell population), enteroendocrine cells (involved in hormone regulation), goblet cells (secreting mucin glycoproteins), M cells (capturing antigens), and Paneth cells (secreting antimicrobial peptides) (Álvarez et al., 2021). Paneth cells play a critical role in selectively controlling GIT-resident bacteria by producing peptides that interact with specific pathogens (Yu et al., 2020).

Among the antimicrobial compounds secreted by Paneth cells are α-defensins, cryptdin-related sequences, REG3α, and enzymes like sPLA2, ANG4, and lysozyme-C. These peptides and enzymes are vital for combating bacterial pathogens, exhibiting antimicrobial activity against both Gram-positive and negative strains (Bevins and Salzman, 2011; Yang and Shen, 2021).

Defensins, a well-known peptide family, are key in antimicrobial defense. These peptides typically contain 30–40 amino acids and six cysteines that form disulfide bonds, stabilizing their three-dimensional structure (Ganz, 2003; Ouellette, 2011; Zhao and Lu, 2014).

During the final stages of the inflammatory process, cytokines are released to assist defense mechanisms of the body. The production of these cytokines is triggered by the presence of antigens, leading to a range of effects on the body’s inflammatory response. Cytokines play a crucial role in defending against infections and maintaining cellular homeostasis (Liu et al., 2021b). These molecules are classified into various groups, each with distinct mechanisms and functions in immune responses. In this context, measuring cytokine levels can serve as an important tool for assessing the extent of infection within the body (Deets and Vance, 2021).

The biological activity and concentration of each cytokine vary depending on the specific disease or infectious agent involved (Liu et al., 2021b). For instance, interferons (IFN) such as IFN-α and IFN-β are activated when cells are infected by viruses or parasites. These interferons function by degrading viral mRNA, inhibiting protein synthesis, and blocking viral replication. Conversely, IFN-γ acts as an immunomodulator, enhancing macrophage activation and promoting the T lymphocyte response profile (Kak et al., 2018; Deets and Vance, 2021; Wang et al., 2021).

The TNF superfamily of ligands and receptors plays a critical role in immune defense against tumors, infections, and autoimmune reactions by promoting cell survival, proliferation, and programmed cell death (Vanamee and Faustman, 2018). These pathways are essential for maintaining the balance between immune activation and regulation, ensuring effective responses while preventing excessive tissue damage.

Furthermore, ILs are pivotal immunomodulatory molecules that regulate the activity of various immune cells. For instance, IL-1 is a potent pro-inflammatory cytokine that triggers inflammation in response to infection or injury (Yazdi and Ghoreschi, 2016), while IL-2 promotes the proliferation of T-cells, which are crucial for adaptive immunity (Malek, 2008). Interleukins such as IL-4, IL-5, and IL-6 are involved in allergic responses and the defense against parasites, whereas IL-10, IL-13, and IL-19 act to suppress immune responses, helping to prevent overactivation and tissue damage (Liu et al., 2021b).

## Gut Microbiota and Disease Development

### Inflammatory bowel diseases

The homeostasis of the intestinal microbiota is directly linked to the response of the individual’s innate and adaptive immune systems. This balance is crucial for maintaining both pro-inflammatory and anti-inflammatory responses. However, factors such as genetic disorders, infectious agents, immunological and neurological defects, age, gender, environmental influences, geographic location, drug intake, and inflammatory components in the diet can disrupt microbiota, negatively impacting mucosal health and immune function (Forbes et al., 2016; Rohr et al., 2018).

Inflammatory bowel disease is a debilitating condition that leads to chronic inflammation and pain, potentially impacting a patient’s quality of life. While it does not directly affect life expectancy, the chronicity and broad range of symptoms can significantly diminish life quality. The chronic nature of the disease is characterized by three stages: (1) penetration of components from the lumen into the lamina propria; (2) failure to eliminate the material within the lamina propria; and (3) compensation by the adaptive immune response (Sewell et al., 2009). Within this spectrum, CD and UC are two primary forms of IBD (Rohr et al., 2018).

CD is an idiopathic disease characterized by inflammation of the GIT, affecting regions from the mouth to the anus (Petagna et al., 2020). This disease involves inflammation in the lamina propria and may develop into granulomas, although their absence does not rule out the diagnosis. While the exact mechanisms remain unclear, genetic factors such as Muc2 polymorphisms, which affect mucus production and increase susceptibility to bacterial invasion, and alterations in FUT2, which reduce antigen secretion and enhance bacterial interaction with the tissue, are implicated in the development of CD (Petagna et al., 2020). Both innate and adaptive immune responses, including the activation of macrophages, neutrophils, and T cells, contribute to the pro-inflammatory state of this disease, leading to the release of cytokines such as interferon-gamma, IL-2, IL-12, IL-18, and TNF-α (Petagna et al., 2020; Ranasinghe et al., 2025).

UC, conversely, is another idiopathic, relapsing-remitting disease within the IBD spectrum, characterized by inflammation that primarily affects the distal portion of the colon and may extend to the entire colon (Segal et al., 2021). While UC shares similarities with CD, it presents distinct genetic predispositions, risk factors, clinical manifestations, and histological features. Mucosal immune dysregulation, particularly in patients genetically predisposed to the condition, leads to gut inflammation that remains confined to the superficial mucosa (Ordás et al., 2012). Like CD, microbiota imbalance, such as Muc2 polymorphisms, triggers macrophage and antigen-presenting cell activation, leading to a cascade of inflammatory responses. The migration of immune cells, including T cells and natural killer cells, further exacerbates the inflammation, with the secretion of cytokines such as IL-6, IL-9, IL-12, IL-13, IL-26, and IL-36 (Kobayashi et al., 2020).

Beyond inflammatory diseases, metabolic disorders are also linked to microbiota imbalances and inflammation. When the microbiota composition is disrupted, it can contribute to conditions such as obesity, type 2 diabetes, lipid disorders, and other metabolic syndromes (Karlsson et al., 2013). The microbiota plays a significant role in regulating enzymes, metabolic, and biochemical processes, assisting in energy metabolism, and is often considered a metabolically active organ (Pascale et al., 2018).

In obese patients, differences in microbiota composition are observed, with a reduction in Bacteroides bacteria and an increase in *Bacillaceae*, *Clostridiaceae*, and *Firmicutes* phyla. This dysbiosis, which is linked to an unhealthy lifestyle and poor diet, is a key factor in obesity development. Studies with germ-free and colonized mice have shown that the latter eat less food, demonstrating that higher food intake alone does not explain the obese phenotype. Additionally, activation of AMP-dependent protein kinase and expression of angiopoietin-like protein 4 (also known as fasting-induced adiposity factor) are linked to lower energy expenditure and suppressed by microbiota, highlighting its potential influence on obesity (Karlsson et al., 2013; Pascale et al., 2018).

In type 2 diabetes patients, metagenomic studies revealed a decreased abundance of butyrate-producing bacteria, which are known to have anti-inflammatory effects, in comparison to healthy individuals. Additionally, changes in *Faecalibacterium prausnitzii* populations were observed in bariatric patients, where the abundance of these bacteria increased following surgery. Transplantation of microbiota from healthy donors into insulin-resistant patients improved insulin sensitivity and enhanced levels of butyrate-producing bacteria (Karlsson et al., 2013; Parada Venegas et al., 2019; Siddiqui and Cresci, 2021).

Metabolic issues linked to microbiota imbalances also extend to lipid metabolism. The microbiota influences bile acid metabolism, and when disrupted, it can impair the formation of micelles, affecting lipid digestion and absorption. In obese patients, dysregulated bacteria produce fewer secondary bile acids, leading to an abundance of primary bile acids, which can promote the emulsification and absorption of lipids (Woting and Blaut, 2016).

Maintaining the balance of the intestinal microbiota, a complex ecosystem of microorganisms, is crucial for the host’s health. It not only supports essential nutrient production, such as vitamins K and B complex, but also contributes to cellulose digestion and other vital enteric functions. The stability of metabolic processes is closely tied to this microbial equilibrium (Pascale et al., 2018).

## Gut–Brain Axis: Gastrointestinal Pathology and Neurological Diseases

The gut–brain axis functions as a bidirectional communication pathway linking the gut and the brain, playing a crucial role in maintaining gastrointestinal homeostasis and modulating emotions, motivation, and cognitive processes (Carabotti et al., 2015). Although metabolic and immune pathways contribute to the interaction between the microbiome, gut, and brain, signaling mediated by the vagus nerve remains the most direct and efficient route for the gut microbiota to influence brain function (Lee et al., 2024a). This system links intestinal mechanisms, such as immune activation and enteroendocrine signaling, to the central nervous system (CNS), the autonomic nervous system, and the hypothalamic-pituitary–adrenal (HPA) axis (Rusch et al., 2023). The HPA axis is activated in stressful situations, leading to the release of cortisol, which affects both the brain and intestinal cells (Carabotti et al., 2015).

This interconnected system emphasizes the importance of the body–mind relationship in maintaining gastrointestinal homeostasis. Recent research highlights the crucial role of the gut microbiota in this axis. Microbial products and metabolites produced by gut microorganisms act as signaling molecules, exerting direct or indirect effects on the CNS and ENS (Mitrea et al., 2022). Moreover, the gut microbiota is capable of synthesizing important neurotransmitters, such as acetylcholine, histamine, norepinephrine, dopamine, gamma-aminobutyric acid (GABA), and serotonin (Dicks, 2022). Serotonin, for example, is predominantly synthesized in the gut, where it regulates important functions such as motor and secretory reflexes, in addition to acting on platelets, the immune system, bones, and the heart (Yano et al., 2015). Platelets sequester serotonin from the gut, promoting hemostasis and distributing it throughout the body. Dysregulation of peripheral serotonin is linked to diseases such as irritable bowel syndrome, cardiovascular disorders, and osteoporosis (Gros et al., 2021). Understanding these mechanisms is essential for the development of effective treatments.

The gut microbiome has gained recognition as an influential factor in neurodegenerative diseases. Early alterations in the microbiome have been identified in patients with preclinical Alzheimer’s disease (AD) and in individuals with prodromal Parkinson’s disease (PD) (Ferreiro et al., 2023).

Studies in animal models provide strong evidence that these microbiota alterations play a key role in the pathogenesis of neurodegenerative diseases (Loh et al., 2024). This effect occurs primarily through the modulation of microglial functions and activation, a key immune cell in the central nervous system. Microglial activation and neuroinflammation are common pathological features in neurodegenerative diseases (de Araújo Boleti et al., 2020).

The gut–microbiota–brain axis emerges as a crucial regulator of glial functions, positioning itself as a promising target for therapeutic interventions that could delay or prevent the development and progression of these diseases (Loh et al., 2024). These findings reinforce the relevance of microbiota as a modulator of both brain and intestinal functions, expanding our understanding of the complex interactions between body and mind.

### Microbiota and anxiety

Anxiety is often associated with a decline in quality of life and the development of various neurological disorders, such as depression, stress, and obesity. It can be triggered by stressful situations in daily life, such as work environments (Stout et al., 2013), relationships (Porter and Chambless, 2017), studies (Damayanti and Listyani, 2020), or even unavoidable fears, such as the fear of death (Neimeyer and Van Brunt, 2018), the coronavirus pandemic (Coelho et al., 2020), and aging (Rittenour and Cohen, 2016).

In recent years, anxiety has been investigated in relation to dysbiosis of the gut microbiota, especially when compared to a healthy and normal microbiota, which is rich in *Bacteroides* and *Firmicutes*, with the presence of *Actinobacteria*, *Fusobacteria*, *Proteobacteria*, and *Verrucomicrobia* (Eckburg et al., 2005; Qin et al., 2010). Anxious patients show a decrease in bacterial taxa such as *Bacteroidetes*, *Ruminococcus gnavus*, and *Fusobacterium* (Jiang et al., 2018).

In contrast, healthy patients display a gut microbiota that is richer in genera such as *Faecalibacterium*, *Eubacterium rectale*, *Sutterella*, *Lachnospira*, and *Butyricicoccus*, which are less common in anxious patients (Jiang et al., 2018). Patients with generalized anxiety disorder exhibit an enrichment of certain genera. When in a remissive state, they show a decrease in *Bacteroides spp.*, while the three genera (*Sutterella*, *Faecalibacterium*, and *Eubacterium rectale*) that produce SCFAs are more prevalent in the remissive state than in the active state of the illness (Jiang et al., 2018).

The dysbiosis of the gut microbiota, including both Gram-positive and negative bacteria, has been shown to be associated with the development of cancer (Zou et al., 2018; Niedzwiedz et al., 2019), arthritis (Horta-Baas et al., 2017), and inflammatory markers (Salguero et al., 2019), which can lead to alterations in intestinal permeability (Foster and McVey Neufeld, 2013; Simpson et al., 2020; Simpson et al., 2021).

These circumstances can escalate gut inflammation to neuroinflammation through the invasion of cytokines, immune cells, and neurotransmitters into the CNS via the visceral afferents and the vagus nerve. Neuroinflammatory-related changes in the CNS can serve as biomarkers for anxious-like behavior and anxiety (Borovikova et al., 2000; Simpson et al., 2020, 2021; Won and Kim, 2020).

The microbiota can also regulate stress responses (Dinan and Cryan, 2012), and stress can also be a major inducer of anxiety. Chronic stress in germ-free 6-week-old male Kunming mice can increase the release of aldosterone, corticotropin-releasing hormone, cortisol, and adrenocorticotropic hormone in the HPA axis (Huo et al., 2017). The stress-related hormone corticotropin-releasing hormone is also linked to the synaptic input of the locus coeruleus, which, under acute stress, releases noradrenaline and induces a state of anxiety. Acute stress–induced anxiety can be triggered in the basolateral amygdala through adrenergic receptor activation (Daviu et al., 2019).

The total absence of microorganisms in the gut can also be problematic, as it may induce anxious-like behavior in germ-free female Swiss Webster mice tested in the elevated plus maze. These mice also show higher levels of corticosterone compared to conventionally reared specific pathogen-free mice (Walf and Frye, 2007; Neufeld et al., 2011). Similarly, germ-free F344 male rats exhibit a neuroendocrine and behavioral response, with stress-related hormones in the HPA axis, in response to open-field stress (Crumeyrolle-Arias et al., 2014).

Diet can also impact gut microbiomes, which in turn can affect anxious behavior. For example, magnesium deficiency in C57BL/6 mice causes anxiety-like behavior, as indicated by their aversion to light when tested in the light/dark box anxiety test (Poleszak et al., 2004; Pyndt Jørgensen et al., 2015).

The rates of comorbidity for generalized anxiety disorder are very high and often lead to other anxiety and depressive disorders, such as panic disorder, social anxiety disorder, persistent depressive disorder, and major depressive disorder (Noyes, 2001; Beesdo et al., 2010; Lamers et al., 2011). Anxiety can also be a comorbidity resulting from other illnesses associated with gut dysbiosis and the inflammatory-neuroinflammatory nature of conditions such as cancer (Soares et al., 2019), obesity (Alonso-Caraballo et al., 2019; Amiri and Behnezhad, 2019), and depression (Lamers et al., 2011).

In adult male BALB/c mice fed orally with *L. rhamnosus*, modulation of the mRNA expression levels of GABAAα2, GABAAα1, and GABAB1b receptors was observed. This modulation affects receptors implicated in anxiety within the GABAergic system via the vagus nerve in the hippocampus, showing beneficial effects in depressive-related behavior as assessed by the forced swim test (Bravo et al., 2011; Chen et al., 2019; Costa, 2019; Felice et al., 2020).

Probiotic treatment can regulate the endocannabinoid system. *L. acidophilus* has been shown to influence the expression of cannabinoid receptors in the intestines of rodents (Rousseaux et al., 2007), while *Akkermansia muciniphila* increases endocannabinoid levels (Everard et al., 2013). The improved endocannabinoid system helps regulate gut permeability and plasma level of LPS (Muccioli et al., 2010; Batista et al., 2019).

### Microbiota and Alzheimer’s disease

According to AD International, dementia is a group of signs and symptoms that affect the nervous system, leading to the deterioration of an individual’s cognitive abilities, including memory and vital functions. The most common type of dementia, which affects about 50%–60% of people diagnosed worldwide, is AD. However, AD can be confused with other forms of dementia that share similar characteristics, particularly memory loss and emotional changes. These include vascular dementia, frontotemporal dementia, and Lewy body dementia (Koenig et al., 2018).

The pathophysiology of AD is primarily characterized by the presence of amyloid-β peptides, which form plaques in the cerebellar amygdala and the entorhinal cortex of the temporal lobe, along with TAU protein, which forms intracellular neurofibrillary tangles. These compounds are widely observed in patients with AD and other dementias. Their presence triggers diffuse cortical atrophy, as well as neurovascular and synaptic degeneration, which impact the response of various neurotransmitters, leading to neuroinflammation (Thaiss et al., 2016; Wells et al., 2017).

The origin of AD remains uncertain; however, it is speculated that its etiology is linked to both genetic and environmental factors. This highlights the importance of observing an individual’s quality of life and lifestyle, as these factors may contribute to the potential diagnosis of some form of dementia (Jiang et al., 2017).

It is known that the main risk factors associated with AD are advanced age, genetic predisposition, and preexisting vascular diseases. However, some studies suggest that, as with other pathologies, AD may also be linked to changes in intestinal microbiota of an individual, as the microbiota can influence brain function. In this context, it is noted that diet of an individual throughout life may act as a risk factor for the onset and progression of the disease (Jiang et al., 2017).

A study conducted with transgenic and wild-type mice aimed to analyze and compare the intestinal microbiota of individuals with AD throughout the disease and healthy individuals. The researchers found that microbiota of AD mice changed during disease progression, with some microorganisms no longer being identified. In contrast, the microbiota of the wild-type mice did not show significant changes (Kuang et al., 2019).

Other studies have shown that alterations in the microbiota are linked to the neuroinflammatory signs characteristic of AD. The researchers concluded that, as the disease progresses, there is activation of microglia. It was observed that the M1 type of microglia, which is pro-inflammatory, increased in the later stages, while the M2 type, which is neuroprotective, decreased throughout the disease. In addition to microglia, an increase in the NLRP3 inflammasome was also observed, supporting the hypothesis of a relationship between the microbiota and neuroinflammation (Bevins and Salzman, 2011; Ouellette, 2011; Yang and Shen, 2021).

In addition to microglial activation, the researchers also observed peripheral infiltration of immune cells in the brain, with certain cell types being more abundant in individuals with AD. Like M1 microglia, Th1 cells were also abundant; as the microbiota changes, this type of cell increases during disease progression. The study concluded that the gut microbiota is associated with peripheral infiltration of immune cells into the brain, microglial activation, and subsequently induced neuroinflammation (Wang et al., 2019).

Gut microbiome modulates neuroinflammation in AD by regulating microglial activation states via microbial metabolites. For instance, *Akkermansia muciniphila*-derived propionate has been shown to suppress amyloid-β–induced microglial hyperactivation (M1 phenotype) while promoting anti-inflammatory (M2) responses, thereby preserving myelin integrity (Vogt et al., 2017; Colombo et al., 2021). Conversely, dysbiosis-associated increases in LPS-producing bacteria (e.g., *Escherichia coli*) have been demonstrated to exacerbate neuroinflammation and oligodendrocyte dysfunction, thereby accelerating white matter damage in AD (Xu et al., 2023). These mechanisms suggest that the composition of gut microbiota exerts a direct influence on glial reactivity and demyelination in neurodegenerative pathologies.

A study conducted with 89 older adults found an association between the presence of pro-inflammatory mediators, such as cytokines, and AD (Marizzoni et al., 2020). Another study, which examined changes in the intestinal microbiota from a genetic perspective, observed an increase in the *Blautia* genus, which appeared to have a protective effect against AD. The study also found that a low abundance of the *Erysipelotrichaceae* family, *Erysipelotrichales* order, and *Erysipelotrichales* class was associated with the development of AD, while a greater abundance of the *Porphyromonadaceae* family was observed in affected individuals (Zhao and Lu, 2014; Liu et al., 2021a).

Some studies have shown that the gut microbiota in AD is unique, with observed changes in the presence and decline of bacterial colonies throughout the disease. These studies compared the bacterial composition of a control group with that of AD patients at the levels of phylum, class, family, genus, and species. They found changes in the concentrations of specific microorganisms and an imbalance in the microbiota in AD, particularly in relation to cognition and the deposition of amyloid-β plaques in the brain (Zhao and Lu, 2014; Kak et al., 2018; Vanamee and Faustman, 2018; Deets and Vance, 2021; Wang et al., 2021).

The relationship between intestinal microbiota and AD was further confirmed in an experiment with modified ADLP^APT^ and wild-type mice. The mice were subjected to genetic analysis at different stages of life, revealing that the intestinal microbiota of affected mice was altered even before the onset of the disease, at an early age. The researchers also reported that the presence of microbial components, such as LPS, in the altered microbiota might be linked to the pathogenesis of the disease (Ganz, 2003; Yazdi and Ghoreschi, 2016).

Interventions targeting the gut-brain axis, such as SCFA supplementation or Bifidobacterium breve administration, have demonstrated efficacy in reducing microglial activation and enhancing remyelination in AD rodent models (Marizzoni et al., 2020; Bonfili et al., 2021). The method of fecal microbiota transplantation (FMT) has been studied as a potential treatment for AD. In APP/PS1 mice, FMT from healthy donors not only improved cognitive function but also reduced amyloid-β deposition and microglial activation, correlating with increased SCFA-producing bacteria and decreased pro-inflammatory cytokines (Sun et al., 2019). Further studies are needed to elucidate specific microbial strains and metabolites that optimize glial homeostasis and myelin repair in AD.

The method of FMT has been studied as a potential treatment for AD. The effectiveness of this approach was observed in several studies, which concluded that exposing AD mice to FMT from healthy individuals resulted in improvements in cognition, including memory and fear perception. Additionally, FMT led to a reduction in amyloid-β plaque deposition, TAU protein phosphorylation in the mouse brain, and decreased microglial activation (Malek, 2008; Forbes et al., 2016). In another study, transferring the fecal microbiota from AD mice to healthy mice resulted in significant changes in the gut microbiota, further confirming the importance of the gut microbiota in the pathogenesis of AD (Kim et al., 2021b).

The use of probiotics in controlling AD in mice has shown positive effects, including the improvement of protective metabolites that enhanced cognitive function in the tested animals. These improvements were observed in memory, as well as in reducing signs of anxiety and obsessive behaviors. Additionally, probiotics led to a reduction in the deposition of amyloid-β in the cortex, hippocampus, and amygdala, and provided protection against the release of harmful microbial compounds such as LPS. This, in turn, helped control and reduce neuroinflammation (Sewell et al., 2009; Rohr et al., 2018; Barker and Weiss, 2019).

### Microbiota and Parkinson’s disease: Fecal transplant

PD is a common neurodegenerative condition among the elderly, characterized by tremors, muscle rigidity, akinesia, and postural instability. The primary pathological features of PD include the accumulation of α-synuclein and the progressive degeneration of dopaminergic neurons in the substantia nigra (Radad et al., 2023). The incidence of PD is associated with age, genetic and environmental factors, and lifestyle, but its etiology remains incompletely defined. Its pathogenesis is complex, involving mechanisms such as mitochondrial dysfunction, cellular transport issues, and neuroinflammation (Henrich et al., 2023).

Recent studies indicate that gut microbiota is linked to the development of human diseases, including PD. The gut–microbiome–brain axis is a connection between the gastrointestinal tract, its microbiome (bacteria, yeasts, and viruses), and the CNS (Chaudhry et al., 2023).

It is a complex communication network that influences the gastrointestinal tract, movement, and advanced cognitive and emotional functions. This communication occurs through enteric reflexes, immune activation, enteroendocrine signaling, and changes in intestinal permeability (Aljeradat et al., 2024).

The α-synuclein could be transmitted from the gut to the brain via the vagus nerve, leading to motor symptoms of PD. These studies highlight the significance of the gut–brain axis in the progression of PD (Holmqvist et al., 2014). Furthermore, a significant difference in gut microbiota has been observed between individuals with PD and healthy controls, with correlations identified between key microbiota categories and PD symptoms (Aho et al., 2019).

Currently, the main treatment for PD is dopamine replacement, which alleviates motor symptoms but does not affect non-motor symptoms. Although levodopa has been shown to reduce amyloid-β pathology in AD models, there is no evidence that it reduces α-synuclein deposition in PD (Lee et al., 2024b). Given the relationship between the gut microbiota and PD, new therapies such as FMT have been explored. FMT can restore the gut microbiota, improve the brain-gut axis interaction, and provide neuroprotective effects, potentially improving both motor and non-motor symptoms of PD (Shekar et al., 2025).

The pilot study conducted by DuPont et al. (2023) assessed the effects of fecal microbiota transplantation FMT in patients with PD. The main findings showed that FMT was well tolerated, with transient and mild gastrointestinal symptoms. Intestinal microbiome diversity significantly increased after FMT treatment, particularly regarding the phylum Firmicutes, while Proteobacteria were reduced. Although no objective improvement in motor symptoms was observed, patients who received FMT reported subjective improvements, including reduced constipation and improved intestinal transit and motility. The data suggest that FMT had beneficial effects on the gut microbiota and improved some non-motor symptoms of PD, particularly constipation (DuPont et al., 2023). A reduced abundance of Prevotella may be linked to accelerated disease progression (Aho et al., 2019).

The gut microbiota of 80 PD patients and 77 controls was analyzed to explore its relationship with clinical symptoms and plasma cytokine levels. The study identified an increase in Verrucomicrobia and Bacteroides in PD patients, while Prevotella was more abundant in controls. A significant correlation was found between Bacteroides abundance and motor symptom severity. Furthermore, PD patients showed elevated plasma levels of TNF-α and IFN-γ, indicating abnormal immune responses. These findings suggest that alterations in gut microbiota and inflammation may play critical roles in the progression of PD (Lin et al., 2019).

### Microbiota and depression

Depression is a significant global public health concern, affecting approximately 3.8% of the world’s population, or around 280 million people. This disease poses a serious public health challenge due to its difficult diagnosis and is often confused with other neuropsychological disorders. Depression also carries the harsh reality of social stigma and treatment difficulties, particularly in low-income countries. It has been identified as one of the leading causes of suicide among young people aged 15 to 29 years worldwide (Yokoya et al., 2018).

Similar to other neuropathology, several studies have pointed to a relationship between depression and intestinal dysbiosis. It is also important to note that depression can coexist with other conditions, such as AD, obesity, lipodystrophies, schizophrenia, and inflammatory gastrointestinal diseases (Petagna et al., 2020; Segal et al., 2021). The microbiota–brain–gut connection has been increasingly explored in recent years, with numerous studies demonstrating how the proper functioning of the gut microbiota influences neurological disorders. It was observed that patients with depression treated with probiotics showed significant improvements in their condition, along with an increase in beneficial microorganisms (Ordás et al., 2012; Kobayashi et al., 2020). Another study found that a low-carb diet in individuals with type 2 diabetes and depression resulted in improved depressive symptoms, as well as an increase in beneficial bacteria and SCFAs (Karlsson et al., 2013; Segal et al., 2021).

UC is a disease that can be associated with depression and anxiety. Studies examining the relationship between the microbiota of individuals with colitis and conditions such as depression, anxiety, and intestinal dysbiosis have shown a low diversity of microorganisms. The inflammatory state in these patients may be directly linked to the signs and symptoms of depression, suggesting that addressing dysbiosis could help manage depression (Yuan et al., 2021).

A genetic study has revealed a link between gastrointestinal inflammatory diseases and genetic predisposition, as well as their relationship with the development of depressive disorder. In this study, eight depression phenotypes were analyzed, and all were found to be associated with comorbidities such as peptic ulcers, gastroesophageal reflux, irritable bowel syndrome, and inflammatory bowel disease. These conditions were further linked to the presence of the pathogen Helicobacter pylori, a bacterium involved in gastrointestinal diseases like gastritis (Wu et al., 2021). Depression and CD are closely related, as evidenced by differences in macrophage composition between patients with and without depression. The M1 macrophage phenotype, known for its inflammatory response, has been previously linked to gut inflammation (Tang et al., 2020).

### Microbiota and autism spectrum disorder

Early infantile autism was first clinically described by Leo Kanner in 1943 (Kanner, 1968). Since then, autism spectrum disorder (ASD) has been recognized as a group of neurodevelopmental disorders with multifactorial etiology, characterized by deficits in communication and social interactions (Hodges et al., 2020), as well as restricted interests and repetitive behaviors (Supekar et al., 2021). The incidence of ASD cases is increasing, with a global prevalence estimated at 1%, and this number continues to grow, with a financial impact of approximately US$ 3.5 million per person (Patrick et al., 2021). Clinical diagnosis is conducted by specialized professionals and requires significant effort. While the average age of ASD diagnosis is around 6 years, growing evidence suggests that it can be diagnosed as early as 3 years. This delay in diagnosis has led to higher morbidity and reduced utilization of neuronal plasticity during the critical early years of life.

Despite increased visibility and understanding of the multivariate aspects of ASD including associations with genetic factors (Kim and Leventhal, 2014), immune responses (Björklund Å et al., 2016), and maternal exposure to infections (Zerbo et al., 2013) and pollutants during pregnancy (Traglia et al., 2017), there are still many uncertainties about the full range of factors responsible for the complexity of behavioral and cognitive changes in these disorders. However, the etiology of ASD extends beyond neurological development. Gastrointestinal issues, such as constipation, diarrhea, and abdominal pain, have been commonly reported among individuals with ASD, even in childhood (Cuffman and Burkhart, 2021). Additionally, there is a significantly higher prevalence of food allergies and gastrointestinal inflammation in individuals with ASD compared to typically developing children (Xu et al., 2018).

What is known is that, despite the unique signs and cognitive impairments exhibited by each individual, all of these factors are linked to widespread physiological changes, including alterations in the composition of the gut microbiota. In this context, children with ASD often experience sensory processing issues, which in turn lead to increased food refusal (Kim and Leventhal, 2015; Patrick et al., 2021). This can directly impact the gut microbiota and contribute to the development of eating disorders.

Growing evidence suggests that emerging knowledge of the microbiota and its metabolic consequences are potential triggers for ASD and play a critical role in its etiopathogenesis (Oh and Cheon, 2020). Several studies indicate that children with ASD exhibit a disturbed microbiota composition, accompanied by altered production of bacterial metabolites, when compared to neurotypical children (Zerbo et al., 2013; Traglia et al., 2017; Cuffman and Burkhart, 2021). It has long been believed that any imbalance in the gut microbiota can affect the gut–brain axis during critical periods of a child’s development (Averina et al., 2020).

In this context, the gut microbiota can communicate bidirectionally with the brain. Understanding the relationship between brain function and gut microbiota composition is one of the most fascinating areas of microbiome research. A previous study has reported that microbial metabolites, primarily butyrate and propionate, can contribute to ASD symptoms by influencing the immune system due to their neuroactive properties (Mirzaei et al., 2021). Given that these SCFAs produced by gut bacteria impact the gut–brain axis, the major bacterial phyla identified in maintaining individual homeostasis include *Bacteroidetes*, *Firmicutes*, *Proteobacteria*, and *Actinobacteria*.

In recent decades, gut microbiota dysbiosis has been reported in individuals with ASD compared to typically developing control subjects (Oh and Cheon, 2020; Petitpierre et al., 2021; Canals-Sans et al., 2022). These frequent alterations in the composition and diversity of the gut microbiota, though the results remain controversial, have shown negative effects on host health. To obtain conclusive results, researchers have accumulated evidence from both human and animal studies, which exhibit higher levels of potentially harmful bacteria, such as *Clostridium*, and lower counts of beneficial bacteria, such as *Bifidobacterium*. These changes may contribute to the etiopathogenesis of ASD (Dan et al., 2020). More recently, Ding and collaborators identified *Erysipelotrichaceae*, *Faecalibacterium*, and *Lachnospiraceae* as potential contributors to ASD impairment, while another study linked an increase in *Desulfovibrio* spp. to the severity of autism, correlating with the intensity of autistic manifestations (Tomova et al., 2015; Ding et al., 2020).

Furthermore, it is important to note that gut dysbiosis refers to shifts in the diversity and/or composition of the gut microbiota. For this reason, dietary intake, genetic factors such as gene mutations and amplifications, as well as epigenetic alterations, could be considered additional elements in the pathogenesis of autism. Given this complexity, all the current knowledge about the role of gut microbiota in ASD must be approached with caution. Under these conditions, there is currently no unique microbial signature that can fully describe the pathogenesis of autism.

Several pieces of evidence have shown that ASD can lead to various language and social difficulties. As a result, significant efforts are being made to explore treatment approaches and therapies aimed at reducing the discomfort and limitations experienced by individuals with ASD (Fuentes et al., 2021).

An important consideration is that ASD, being a developmental disorder that begins in early infancy, has significant and concerning impacts worldwide. This delay in diagnosis hinders the child’s development by making it difficult for them to access specialized interventions, which are crucial for supporting the child’s social, adaptive, and cognitive development (Brentani et al., 2013).

Intense research has focused on the gut microbiota, highlighting its significant role in modulating the host in ASD, as up to 70% of children with ASD experience impaired gastrointestinal function (Gondalia et al., 2012). Given this scenario, the human gut microbiota has become an emerging field with the potential not only to deepen our understanding of ASD, but also to pave the way for revolutionary new therapies based on the identification of mechanisms underlying microorganism-host interactions (Taniya et al., 2022).

Evidence suggests that gut dysbiosis in ASD may influence glial activation and myelination through microbial metabolites. SCFAs, such as butyrate, which are produced by commensal bacteria such as *Faecalibacterium prausnitzii*, have been shown to suppress neuroinflammation by modulating microglial reactivity and promoting oligodendrocyte differentiation (Duscha et al., 2020; Needham et al., 2021). Furthermore, shifts in the composition of the gut microbiota, specifically a decrease in *Bifidobacterium* and *Lactobacillus*, have been shown to be associated with elevated levels of pro-inflammatory cytokines, such as IL-6 and TNF-α (Hsiao et al., 2013; Sharon et al., 2019). These alterations may potentially contribute to the exacerbation of microglial hyperactivation and the impairment of myelin repair in models of ASD (Hsiao et al., 2013; Sharon et al., 2019). These findings underscore the potential significance of the gut microbiome in regulating glial function and white matter integrity in neurodevelopmental disorders.

Encouraged by these findings, the US Food and Drug Administration recognized microbial transplant therapy for ASD treatment in 2019 (Adams et al., 2019). In other words, the involvement of gut microbiota in ASD is compelling. In the future, the US Food and Drug Administration may lead to the development of improved personalized treatments based on suitable bacterial compositions that could help regulate ASD. However, it is important to note that the benefits of gut microbial modulation, including the potential use of probiotics, prebiotics through dietary intake, or FMT, will not cure autism. These are only potential therapeutic applications aimed at reducing autistic manifestations. To date, there is no cure for this neurodevelopmental disorder.

## Obesity and Microbiota

The microbiota is closely linked to metabolic diseases such as obesity, as these bacteria play a key role in maintaining the body’s homeostasis by assisting in lipid metabolism, endocrine functions, immune responses, and energy extraction from food intake, all aspects that are impaired in obesity (Al-Assal et al., 2018). However, despite the beneficial physiologically active substances produced by the microbiota, they can also release potentially harmful substances such as carcinogens, neurotoxins, and immunotoxins (Canfora et al., 2019; Liu et al., 2021a).

Similar to other microbiome-associated conditions, obesity also impacts the diversity of the host microbiota (Heiss and Olofsson, 2018; Ma et al., 2019). Obesity causes an imbalance in the two most abundant bacterial groups in the gut microbiota, *Firmicutes* and *Bacteroidetes*. The increase in *Firmicutes* and the proportional decrease in Bacteroidetes observed in the microbiota of obese individuals can enhance energy harvest from food in obese mice (Ley et al., 2005; Turnbaugh et al., 2006). A similar pattern is seen in obese humans with a high body mass index (Koliada et al., 2017; Indiani et al., 2018).

Obesity can ultimately lead to a lower abundance of *Methanobrevibacter smithii*, *Bifidobacterium animalis*, and *L. plantarum* in the gut microbiota of obese individuals (Schwiertz et al., 2010). Several studies have shown that the relationship between gut bacteria and obesity is species-specific, as different species within the same genus can have opposing effects. For instance, *L. paracasei* is negatively associated with obesity, whereas *L. gasseri* and *L. reuteri* are positively correlated with it (Crovesy et al., 2017), A similar pattern is observed in *Bifidobacterium*, a common probiotic that aids *Bacteroides* in breaking down polysaccharides and inhibiting cholesterol absorption in the small intestine (Pereira and Gibson, 2002; Sonnenburg et al., 2006; Yin et al., 2010).

The gut microbiota can contribute to obesity through various mechanisms, one of which is the increased energy uptake eased by SCFAs produced by bacteria. SCFAs are absorbed and promote adipogenesis and triglyceride storage (Al-Assal et al., 2018; Liu et al., 2021a). Additionally, they are associated with metabolic disorder markers, hypertension, increased gut permeability, and obesity (de la Cuesta-Zuluaga et al., 2018).

The gut microbiota regulates central appetite through multiple mechanisms, including the release of neuromodulators such as serotonin (Shajib and Khan, 2015) and lactate, which serves as a neuronal substrate to prolong satiety after food intake (Silberbauer et al., 2000). It also plays a key role in the gut–brain axis by modulating the secretion of gut hormones by enteroendocrine cells, including vital peptides for gut–brain communication such as glucagon–like peptide-1 and peptide YY (Federico et al., 2016). Dysbiosis of gut microbiota can disrupt these regulatory pathways, leading to increased food intake (Cryan et al., 2019; Liu et al., 2021a).

Increased food intake, particularly in the context of a western diet rich in refined carbohydrates, unhealthy fats, excessive meat, salt, and additives, contributes to obesity and disrupts gut microbiota balance (García-Montero et al., 2021). High-fat diet and high-sucrose diets have been shown to reduce microbial diversity, decreasing the abundance of butyrate-producing bacteria while increasing pathogenic bacteria such as *Alistipes*, *Anaerotruncus*, and *Bacteroides* (Kong et al., 2019).

Fat storage can be regulated by gut microbiota through increased serum glucose levels and enhanced intestinal absorption, leading to the expression of transcription factors such as sterol regulatory element-binding protein-1 and carbohydrate response element-binding protein. These factors promote fat synthesis in the liver, then influence lipid levels in the circulatory system (Bäckhed et al., 2004; Liu et al., 2021a).

Chronic inflammation is a hallmark of obesity and can be worsened by gut microbiota dysbiosis. In obese individuals, microbial imbalance can increase levels of LPS and acetate, leading to elevated serum cholesterol, triggering a pro-inflammatory response, and potentially inducing a cytokine storm. This inflammatory cascade may further aggravate obesity-related comorbidities (Perry et al., 2016; Liu et al., 2022).

The gut microbiota can influence circadian rhythm by regulating the transcription factor NFIL3 and the lipid transporter gene Cd36, thereby affecting lipid uptake, absorption, storage, and obesity (Kuang et al., 2019; Liu et al., 2021a). Specific gut bacteria, including *Bifidobacterium*, *Lactobacillus*, *Bacteroides*, *Clostridiaceae*, *Lachnospiraceae*, and *Ruminococcaceae*, contribute to bile salt biotransformation, an essential process for circadian rhythm modulation (Parkar et al., 2019).

Bariatric surgery is the most common treatment for severe obesity, and it significantly alters the gut microbiota. Post-surgery, there is often an increase in *Akkermansia muciniphila* and modulation of other bacteria such as *Bifidobacterium*, *Lactobacillus*, and *Enterococcus*, depending on obesity severity and the type of procedure performed (Debédat et al., 2019). Given the long recovery period and the need to restore gut microbiota balance, further research is needed on probiotics, prebiotics, specialized diets, and gut microbiota transplants as complementary strategies (Aron-Wisnewsky et al., 2019).

The use of probiotics has appeared as a promising approach to managing obesity by modulating gut microbiota. *Lactobacillus paracasei* has been shown to reduce fat storage in high-fat diet-induced obesity in specific pathogen-free C57B/6J mice by regulating angiopoietin-like 4 protein, a key factor in fat storage regulation (Aronsson et al., 2010). Additionally, a previous study has reported that *E. faecalis, B. longum*, and *L. acidophilus* can help mitigate weight gain in mice-fed high-fat and high-carbohydrate diets (Kong et al., 2019).

FMT from allogenic vegan donors has been shown to induce beneficial changes in gut microbiota, influencing plasma metabolites involved in inflammation and lipid metabolism. Additionally, it modulates hepatic gene expression in obese individuals with nonalcoholic fatty liver disease, such as steatohepatitis (Witjes et al., 2020).

## Gut Microbiota and Skin

Although distinct organs, the gut and the brain show similarities in features and functionality, both playing crucial roles in immune and neuroendocrine functions. They are highly innervated and vascularized, and covered by epithelial cells that form a vital link between the internal body and the external environment, serving as a first line of defense (Thoo et al., 2019; De Pessemier et al., 2021).

The intestinal microbiota plays essential roles from the beginning of life, including defending the host against pathogens, supporting metabolic activities, helping in the degradation of drugs and toxins, and synthesizing vitamins. It also has beneficial impacts on the physiology and homeostasis of gut and skin tissues. Any alteration in the gut microbiota can influence skin health through its metabolic activity and immunological effects (Funk et al., 2020; Hou et al., 2022).

The skin is the largest organ of the human body, serving as a physical, chemical, and immunological barrier between the external environment and the internal body. In addition to providing protection, it plays a crucial role in water retention and temperature regulation. The skin undergoes constant epidermal turnover, a process of continuous cell renewal. Epidermal cells originate from stem cells and differentiate into three types, basal, spiny, and granular cells, before forming the outermost layer of the epidermis, the stratum corneum (Swaney and Kalan, 2021). When epidermal turnover functions properly, the epidermis maintains an effective skin barrier, preventing excessive water loss, conserving skin moisture, and protecting against the invasion of microorganisms and other harmful substances (Abhishek and Palamadai Krishnan, 2016; Gaur et al., 2017).

Healthy human skin hosts a diverse microbial ecosystem, with each region supporting a distinct microbial composition. For example, moist areas are predominantly colonized by *Corynebacteria* and *Staphylococci*, while oily regions are primarily inhabited by *Propionibacterium* (Grice et al., 2009). Culture-independent methods have revealed that healthy skin harbors over 1000 bacterial species, with the most common genera including *Brevibacterium*, *Propionibacterium*, *Micrococcus*, *Staphylococcus*, *Streptococcus*, and *Corynebacterium*. Additionally, the predominant fungal genus found on the skin is *Malassezia*. Under healthy conditions, these microbial communities interact with the host in both parasitic and commensal relationships (Coates et al., 2019).

The gut–skin axis refers to the bidirectional relationship between the gut and the skin. The mechanisms through which the gut microbiota influences skin homeostasis appear to be linked to its modulatory effects on systemic immunity (Mahmud et al., 2022). Certain intestinal metabolites and microorganisms, such as *Bacteroides fragilis*, *Faecalibacterium prausnitzii*, and *Clostridium spp.*, promote the accumulation of T lymphocytes and regulatory cells, which aid in anti-inflammatory responses. Conversely, some filamentous bacteria contribute to the accumulation of Th1 and Th17 pro-inflammatory cells (Kosiewicz et al., 2014; Forbes et al., 2016; Salem et al., 2018).

SCFAs synthesized by the intestinal microbiota serve as essential nutrients that support intestinal barrier function through various mechanisms. It is believed that the gut microbiota influences skin microbiota, as SCFAs, specifically propionate, acetate, and butyrate, play a crucial role in shaping the microbial profile of the skin and modulating cutaneous immune defense. Notably, the bacterial genus *Propionibacterium* produces two key SCFAs, acetate and propionic acid, which exhibit potent antimicrobial effects against resistant bacteria such as *S. aureus* (Shu et al., 2013).

Various factors, including the use of antibiotics (Ribeiro et al., 2020), probiotics (Kaur et al., 2020; Kim et al., 2021a), diet (Graf et al., 2015), lifestyle (Martinez et al., 2021), aging (Funk et al., 2020), and disease (Nibali et al., 2014; Zhuang et al., 2020), can influence gut microbiota composition (Rinninella et al., 2019). Disruptions and imbalances in microbial strains may significantly impact overall skin homeostasis, potentially leading to inflammation and dermatological conditions such as *Acne vulgaris*, atopic dermatitis, and psoriasis (O’Neill et al., 2016).

### Acne vulgaris

Acne is a chronic inflammatory condition affecting the pilosebaceous unit, clinically presenting as non-inflammatory comedones or inflammatory papules, pustules, nodules, and scarring, primarily on the face and upper trunk. Its pathogenesis is complex and multifactorial, involving increased cutaneous sebum production, sebaceous gland hyperplasia due to hormonal influence, proliferation of *Propionibacterium acnes* strains, and infiltration of inflammatory cells. Acne is particularly prevalent in western countries, where it has been associated with diets high in glycemic load (Zaenglein et al., 2016; Sánchez-Pellicer et al., 2022).

The gut microbiota influences the pathophysiology of acne through its interaction with the serine/threonine kinase mechanistic target of rapamycin (mTOR) signaling pathway. mTOR is a nutrient-sensitive regulator involved in cell proliferation and differentiation processes in the skin, playing a crucial role in homeostasis and the development of an effective epidermal barrier. Additionally, the mTOR pathway regulates gut barrier integrity and can impact gut microbiota composition. In cases of intestinal dysbiosis, compromised gut barrier function may promote metabolic inflammation (Salem et al., 2018). Dysregulation of the mTOR pathway has been linked to various skin diseases, including acne. Studies have reported increased serum insulin-like growth factor 1 concentrations, higher cytoplasmic expression of fork head box nuclear transcription factor-O1, and elevated cytoplasmic and nuclear mTOR expression in acne patients compared to healthy individuals. Furthermore, diets high in glycemic load have been associated with increased serum insulin-like growth factor 1 levels and upregulated fork head box nuclear transcription factor-O1 and mTOR expression (Agamia et al., 2016; Monfrecola et al., 2016; Karagianni et al., 2022).

### Atopic dermatitis

This inflammatory skin disorder is characterized by skin barrier dysfunction, immune dysregulation, genetic predisposition, and environmental factors (Otsuka et al., 2017). Inflammation is primarily driven by the Th2 pathway, with cytokines IL-4 and IL-13 contributing to skin barrier disruption, reduced lipid metabolism, and inhibited antimicrobial peptide synthesis. These conditions create a favorable environment for *S. aureus* overgrowth (Seite and Bieber, 2015). The prevalence of allergic diseases has increased in recent decades, and studies suggest that alterations in the gut microbiota may play a role in this rise (Huang et al., 2017). The gut microbiota is crucial for immune homeostasis, which begins early in life through exposure to maternal microbiota and continues to develop through breast milk and dietary interactions. However, the typical western diet has been shown to negatively impact immune regulation, potentially contributing to the increased prevalence of allergic diseases (Lee et al., 2018).

The western diet is characterized by a high glycemic load and low fiber intake, which alters the gut microbiota and leads to a reduced production of immunomodulatory metabolites, such as SCFAs (Li and Yosipovitch, 2020). SCFAs play a crucial role in anti-inflammatory processes mediated by regulatory T cells and contribute to maintaining epithelial barrier integrity (Maslowski et al., 2009; Biedermann et al., 2015). Dysbiosis-induced reductions in local and systemic immune tolerance may help explain the rising prevalence of autoimmune and atopic diseases (Salem et al., 2018).

Studies have established a link between intestinal dysbiosis and atopic disease. Two Korean studies analyzing fecal metagenomes from patients with AD found a significant reduction in *Faecalibacterium prausnitzii* and decreased SCFA production compared to healthy individuals. The association between intestinal dysbiosis, *F. prausnitzii*, and epithelial barrier dysfunction suggests that compromised gut integrity allows poorly digested food, microbes, and toxins to enter circulation and reach target tissues, including the skin. This process triggers Th2-type immune responses, leading to the release of pro-inflammatory cytokines and exacerbating tissue damage (Kim et al., 2013; Song et al., 2016).

### Psoriasis

Immune-mediated inflammatory disease is among the most prevalent chronic skin conditions worldwide, affecting approximately 0.1%–12% of the population (Parisi et al., 2013; Takeshita et al., 2017). It is a systemic autoimmune disorder characterized by the inappropriate activation of immune-mediated pathways, which leads immune cells to attack the body’s own tissues, resulting in elevated levels of pro-inflammatory cytokines. Psoriasis, a multifactorial disease, can be triggered by various factors such as environmental influences, lifestyle, and genetic predisposition. The clinical manifestation includes red, scaly, and thick skin lesions that may appear anywhere on the body (Caputo et al., 2020; Kierasińska and Donskow-Łysoniewska, 2021).

In patients with psoriasis, a reduction in beneficial microbes can disrupt immune system balance, which in turn affects skin health. Specifically, patients with psoriasis show a decrease in *Bacteroidetes*, *Proteobacteria*, *Actinobacteria*, *Akkermansia muciniphila*, and *Firmicutes* (Scher et al., 2015; Bland, 2016; Zhang et al., 2019). Elevated levels of biomarkers such as claudin-3 and intestinal fatty acid binding protein have also been observed in these patients (Komine, 2020). Bacteroides, for example, produce polysaccharide A, which activates regulatory T-cells and promotes an anti-inflammatory response. Therefore, a reduction in Bacteroides in psoriasis patients can alter the immune response, triggering pro-inflammatory pathways (Mann et al., 2020). Moreover, beneficial microbes in the intestinal barrier produce SCFAs, which help prevent pathogen colonization. A decrease in these beneficial microbes compromises intestinal barrier integrity, allowing bacteria to leak from the gut into the systemic circulation, which may exacerbate psoriasis symptoms (Singh et al., 2017; Mann et al., 2020; Kierasińska and Donskow-Łysoniewska, 2021).

## Clinical Translation Challenges in Microbiota-Directed Therapies

Despite the advancements in gut microbiota research and its impacts on human health and comorbidities, the translation of these findings to effective clinical therapy remains a challenge. One of the barriers is the gut microbiota diversity, which has high variability between individuals. This is impacted by genetics, age, diet, environment, antibiotic exposure, and overall health status (Falony et al., 2016; Zhernakova et al., 2016).

The interindividual heterogeneity significantly affects the reproducibility and efficacy of microbiota-based interventions such as probiotics, prebiotics, and fecal microbiota transplantation (Zmora et al., 2018). Individuals with distinct baseline profiles when subjected to the same dietary intervention may exhibit divergent immunological (levels of proinflammatory marker sCD14 and anti-inflammatory adipose tissue macrophage CD163) and metabolic responses (glucose and insulin concentration in the blood, cholesterol levels, insulin sensitivity), this increases the difficulty to predict clinical trial outcomes across the population (Healey et al., 2017).

Another major translational challenge is the extrapolation of preclinical data, especially in neurogastroenterology. Experimentation with animal models, such as germ-free and gnotobiotic mice, is an important tool to understand the gut-brain axis mechanisms, however, due to differences in the microbiota composition, neuroanatomy, immune system responses, and behavior between animals and humans raises concern when extrapolation preclinical results (Nguyen et al., 2015; Hugenholtz and de Vos, 2018). Moreover, experimenting in rodent microbiota does not fully summarize the complexity and diversity of the human gut ecosystem, leading to potentially misleading conclusions in translational research (Arrieta et al., 2016).

To bridge these gaps in translational research, future research must prioritize and improve humanized microbiota models (Lundberg, 2019), longitudinal clinical studies (Mars et al., 2020), and a multi-omics approach to integrate host-microbiota interactions in a dynamic and personalized manner (Brubaker and Lauffenburger, 2020). Addressing these challenges is essential to achieve the full potential of microbiota-based interventions in gastrointestinal and neurological disorders.

## Conclusion

The rise of numerous multifactorial diseases, including inflammatory, autoimmune, metabolic, neoplastic, and neurodegenerative conditions, many of which are linked to intestinal dysbiosis, compositional and functional alterations of the intestinal microbiome, presents a significant challenge. By connecting the pathogenesis of common diseases to dysbiosis, the microbiome field faces the task of unraveling the mechanisms responsible for the persistence of dysbiosis microbiome configurations. Moreover, it must differentiate between host-microbiome causal relationships and secondary microbial changes that emerge during the progression of disease. Dysbiosis has been implicated in immune disorders, and understanding the causes and consequences of bacterial dysbiosis, as well as its role in the molecular etiology of common diseases, is essential for developing new therapeutic approaches. Gaining a molecular-level understanding of the origins of dysbiosis, its endogenous and environmental regulatory processes, and its downstream effects could pave the way for microbiome-targeted therapies aimed at treating a wide range of immune-mediated diseases.

## Data Availability

*Not applicable.*
